# Tumor-derived SEMA7A regulates fatty acid oxidation in the tumor-associated macrophages to promote the progression of non-small cell lung cancer

**DOI:** 10.3389/fimmu.2025.1625208

**Published:** 2025-09-11

**Authors:** Jia Wang, Yang Shao, Jiameng Zhang, Fangfang Han, Si Wang, Beixing Liu

**Affiliations:** ^1^ Department of Medical Microbiology and Human Parasitology, College of Basic Medical Sciences, China Medical University, Shenyang, China; ^2^ Department of Human Anatomy, Changzhi Medical College, Changzhi, China; ^3^ Department of Laboratory Medicine, Shengjing Hospital of China Medical University, Liaoning Clinical Research Center for Laboratory Medicine, Shenyang, China

**Keywords:** NSCLC, semaphorin 7A(SEMA7A), tumor-associated macrophages, M2polarization, tumor microenvironment

## Abstract

Non-small cell lung cancer (NSCLC) is an aggressive cancer with a poor prognosis. Despite the success of therapies for NSCLC, more investigations of new biomarkers for patient selection are urgently needed. Semaphorin 7A (SEMA7A), a soluble tumor-derived molecule, can modulate the proliferation, invasion and angiogenesis of multiple types of cancers. However, whether SEMA7A contributes to the progression of NSCLC is still unknown. In this study, by using bioinformatics analysis and an experimental murine tumor model, we found that the expression levels of SEMA7A were elevated in the human NSCLC and positively correlated with the poor prognosis. Knockdown of SEMA7A in cancer cells may suppress NSCLC progression, in parallel with a diminished M2 polarization in the tumor microenvironment (TME). In fact, SEMA7A may increase the polarization of tumor-associated macrophages (TAMs) toward the M2 phenotype in an ITGB1-dependent manner. Fatty acid oxidation in macrophages seems to be essential for the ability of SEMA7A to promote M2 polarization. Blockade of fatty acid oxidation may reverse the immunosuppressive phenotype of TAMs and the outcomes of NSCLC. Our findings provide experimental evidence that SEMA7A may act as a regulatory factor for macrophage lipid metabolism, which influences the polarization status of TAMs.

## 1 Introduction

Lung cancer is the most commonly diagnosed cancer and has the highest mortality rate worldwide. There are two main types of lung cancer: non-small cell lung cancer (NSCLC) and small cell lung cancer (SCLC), with NSCLC being the more common type, constituting 85% of total lung cancer cases (1–4). The occurrence of NSCLC is a complex process that requires the involvement of many elements, in which, the soluble molecules released by cancer cells are considered pivotal for inducing immune escape, invasion, and metastasis in NSCLC (5, 6). It has been reported that cancer-secreted soluble synucleins may act as risk factors that stimulate breast cancer invasion and metastasis (7, 8). Therefore, it is important to discover and clarify the tumor-derived molecules to improve diagnosis and therapeutic efficacy of patients with NSCLC.

Semaphorin 7A(SEMA7A), also known as CDw108, belongs to a family of cellular guidance proteins with multiple physiological functions, including neurite growth, bone and immune cell regulation, and inflammation (9–11). These effects of SEMA7A are mediated by two specific receptors, plexin C1 and integrins (12). Recently, studies have revealed that SEMA7A can act as a key player in the tumorigenesis and metastasis of many types of cancers (13). Indeed, SEMA7A has been found to be highly expressed in the tumor cells and tumor tissues of patients with breast carcinoma (14–16). In addition, the expression levels of SEMA7A are positively related to the pulmonary metastasis in a melanoma murine model (17). The tumor-promoting effects of SEMA7A have also been observed in oral cancers and pancreatic cancers (18, 19), suggesting a profound influence of SEMA7A on the development of these cancers. However, whether SEMA7A affects the progression of NSCLC remains unclear.

The tumor microenvironment (TME), which is composed of the extracellular matrix, helper cells, immune cells, chemokines, and growth factors, plays a crucial role in the lung cancer (20, 21). Tumor-associated macrophages (TAMs) are the major cellular components of the TME, and are responsible for the tumor growth, drug resistance, and immune suppression (22, 23). Since M2-polarized macrophages facilitate NSCLC progression and tumor metastasis (24, 25), reprogramming the phenotype of TAMs might be beneficial for the outcomes of NSCLC patients. Considering that macrophages can express the SEMA7A receptor (26, 27), the function and polarization status of TAMs in the TME of NSCLC might be affected by the tumor-derived soluble factor SEMA7A. Previous studies have reported that SEMA7A derived from intestinal epithelial cells can induce IL-10 production by macrophages to ameliorate the severity of colitis (26). Moreover, the interaction between SEMA7A and integrin receptors has been shown to promote macrophage reprogramming into an anti-inflammatory M2 phenotype (28). However, whether SEMA7A might regulate TAM polarization to affect the outcomes of NSCLC is still unknown.

In this study, by using bioinformatics analysis and an established Lewis lung carcinoma mouse model, we found that SEMA7A was upregulated in the patients with NSCLC and was positively correlated with the tumor progression and poor prognosis. Targeting SEMA7A may reduce fatty acid oxidation-dependent M2 polarization in the TME, thereby influencing NSCLC progression.

## 2 Materials and methods

### 2.1 Bioinformatics analysis

The RNAseq data for lung adenocarcinoma (LUAD) and Lung squamous carcinoma (LUSC) were obtained from the Cancer Genome Atlas database (TCGA, https://portal.gdc.cancer.gov/). We extracted the data of SEMA7A expression and then drew a picture to describe the expression pattern of SEMA7A in LUAD and LUSC using R package. We used the tissue samples from the Human Protein Atlas (HPA) database to assess the local expression pattern of SEMA7A in NSCLC. CIBERSORT (a computational tool that uses gene expression data to estimate the abundance of cell types in a mixed cell population) was used to analyze immune cell infiltration in NSCLC from the TCGA databases.

### 2.2 Cell lines and cell culture

The mouse NSCLC cell line and normal lung epithelial cell line MLE-12, as well as human NSCLC cell line H1299 and normal lung epithelial cell line BEAS-2B were purchased from the Cell Bank of the Chinese Academy of Sciences (Shanghai, China). Murine macrophage cell line RAW264.7 was purchased from Fenghui (CL0266; Fenghui Biotechnology, Hunan, China). Cells were cultured routinely in Dulbecco’s modified Eagle’s medium (DMEM, Pricella, Wuhan, China), supplemented with 10% fetal bovine serum (FBS, Pricella, Wuhan, China) and 1% penicillin/streptomycin (Gibco) at 37°C with 5% CO_2_. For coculture experiments, 5×10^5^ of shSEMA7A-transfected cells were seeded in a 6-well plate, followed 5×10^5^ of RAW264.7 cells were seeded onto the membranes of the top chambers of a 0.4-mm pore Transwell chamber (Corning). After incubation for 48 hours, the macrophages were collected for further studies.

### 2.3 RNA interference and lentiviral vector transfection

To silence the expression of SEMA7A in LLC and H1299 cells, specific small interfering RNA (siRNA) targeting mouse and human SEMA7A were designed and synthesized by Sangon Biotech Technologies, Shanghai, China (**Supplementary Table 1**). A total of 3×10^5^ cells were seeded into each well of 6-well plates. After culturing for 24 h, siRNA or negative control (NC) siRNA were transfected into the cells with Lipofectamine 3000 transfection reagent (L3000001; Thermo Scientific, USA) according to the manufacturer’s protocol. Purified lentiviral vectors with puromycin resistance (PLV-puro) for silencing SEMA7A were provided by Sangon Biotech Technologies (PLVE3866, Shanghai, China). After transfection, the stable cells with a silenced target gene were obtained using puromycin selection. The sequences of the designed oligonucleotides against SEMA7A and NC were listed in the **Supplementary Table 1**.

### 2.4 Cell proliferation, invasion and wound healing assay

The Cell-Counting Kit-8 (CCK-8) method was used to analyze the effect of knockdown of SEMA7A on tumor cell proliferation. The migration ability of the NSCLC cells was evaluated via a scratch wound-healing assay after disturbing the expression of SEMA7A in the cells. The role of SEMA7A in cell invasion was detected via Transwell 24-well plates (8 μm wells; Corning).

### 2.5 NSCLC murine model

Specific-pathogen-free (SPF) male C57BL/6J mice (6–8 weeks of age) were purchased from Huafukang Biotechnology (Beijing, China), and maintained with fresh water and autoclaved food at the Laboratory Animal Center, China Medical University. To establish a mouse model of NSCLC, Approximately 1×10^6^ LLC cells or shSEMA7A-LLC cells in 100μl of PBS were implanted subcutaneously into the right axillary region of the mice. Tumor volumes were measured every 2 day, and calculated using the formula: tumor volume=W^2^×L/2, where L represents the longest diameter and W represents the shortest diameter of the tumor. All animal experiments were approved and performed in accordance with the guidelines of the Institutional Animal Care and Use Committee of China Medical University (Approval No. 20240684).

### 2.6 Quantitative real-time PCR

Total RNA was extracted using the TRizol reagents (Vazyme, China) and was converted to cDNA using the Prime Script RT Reagent Kit (RR047A; TaKaRa, Japan) according to the manufacturer’s instructions. Then, PCR was run in a LightCycler^®^ 480 (Roche Molecular Biochemicals) under identical amplification conditions. The results were normalized to β-actin expression and presented as fold change (fold change = 2^−ΔΔCT^). The primer sequences were listed in the **Supplementary Table 2**.

### 2.7 Preparation of single-cell suspension from tumor tissues

Tumor mass were collected on day 21 after LLC implantation, and digested with 2 mg/mL of collagenase D (Sigma) and 100 ng/mL of DNase I (Roche) at 37°C for 1 h. After passing the digested tumor tissue through a 70mm cell strainer, the red blood cells in the single-cell suspension were lysed with 0.15 M ammonium chloride and 1 mM potassium bicarbonate. Then, the cells were washed twice with PBS and were resuspended in RPMI 1640 medium.

### 2.8 Flow cytometry analysis

The single-cell suspension from fresh tumor tissues was incubated firstly with anti-mouse CD16/32 mAbs (BD Bioscience) for blocking the Fc receptor, and stained with anti-mouse fluorescein-conjugated antibodies against surface antigens for 30 minutes on ice. The antibodies used are shown in the **Supplementary Table 3**.

### 2.9 Isolation of macrophages

The single-cell suspension from tumor tissues obtained by 30-70% percoll gradient centrifugation. Single-cell suspension was centrifuged at 300*g* for 10 min at RT. Cell pellets were resuspended in a 30% percoll gradient in a 15 ml polypropylene tube. Take a new 15ml centrifuge tube and add 5ml of 70% percoll separation solution. Use a pipette to slowly add the 30% percoll separation solution containing cells from the previous step into the 70% percoll solution. Gradients were centrifuged without brake for at 500*g* 20 min at 4°C. After centrifugation, cells were collected from the middle layer (5 ml interphase) in the 30–70% percoll gradient. Then, magnetically macrophages were isolated by using F4/80 labelled beads according to the manufacturer’s protocols (130-110-443, Miltenyi Biotec, Nordrhein-Westfalen, Germany). The sorted macrophages were seed into 6-well plates and stimulated with 100 ng/mL of recombinant SEMA7A protein (HEK293; MCE, USA) in the presence or absence of etomoxir (200 μmol/L) (HY-50202; MCE, USA) for 24 h. After incubation, the macrophages were collected for further analysis.

### 2.10 Western blotting analysis

The sorted tumor-associated macrophages and RAW264.7 cells were denatured in RIPA buffer (Beyotime, Shanghai, China). Proteins were separated via SDS-PAGE and were transferred into polyvinylidene fluoride membranes (Millipore, Shanghai, China). After blocked with 5% nonfat milk at room temperature for 2 h, the membranes were incubated with primary antibodies such as Rabbit anti-SEMA7A (1:500; bs-2702R, Bioss), Rabbit anti-ERK1/2 (1:1,000; YT1625, Immunoway) and Rabbit anti-p-ERK1/2 (1:1,000; YP0101, Immunoway) at 4°C overnight. Then, GAPDH were used as loading controls for different protein analysis. The immunocomplexes were visualized with the BeyoECL Plus Chemiluminescent Substrate. Protein band intensity was quantified with Image J software.

### 2.11 ELISA assay

The levels of IL-1β, TNF-α, IL-10 and TGF-β in the tumor tissues were measured via the ELISA kits (Elabscience) according to the manufacturer’s instructions. The absorbance at 450 nm was measured by a microplate reader.

### 2.12 Oxygen consumption rate assay

The sorted macrophages were seeded in 6-well plates (1×10^5^ cells/well) and treated with or without recombinant SEMA7A protein (100 ng/mL) and etomoxir (200 μmol/L) for 48 h. After incubation, the cells were changed to unbuffered assay media, and were incubated in a non-CO2 incubator at 37 °C for 1 h. Oxygen consumption rates (OCR) were measured via an O2k-FluoRespirometer after the sequential addition of oligomycin (1 mmol/L, Sigma), trifluoromethoxy carbonylcyanide phenylhydrazone (1.5 mmol/L, Sigma), and antimycin/rotenone (2 mmol/L, Sigma).

### 2.13 Detection of free fatty acids

Macrophages were sorted from the tumor tissues of the tested mice, and lysed in the extracting solution. The cell lysate was centrifuged at 8000 rpm for 10 minutes before the supernatant was collected. A FFA detection kit (boxbio, China) was used to detect the levels of free fatty acids. The absorbance at 550 nm was measured by a microplate reader.

### 2.14 Statistical analysis

All experiments have been repeated at least three times. Values were presented as mean ± standard deviations (SD). One-way analysis of variance (ANOVA) was used to compare differences between three or more groups. Student’s *t*-test was applied to analyze the differences between the two groups. *p*-values < 0.05 were considered statistically significant. The data were analyzed using GraphPad Prism8 software.

## 3 Results

### 3.1 The expression level of SEMA7A is elevated in the human NSCLC and is positively correlated with the poor prognosis

**Figure 1:**
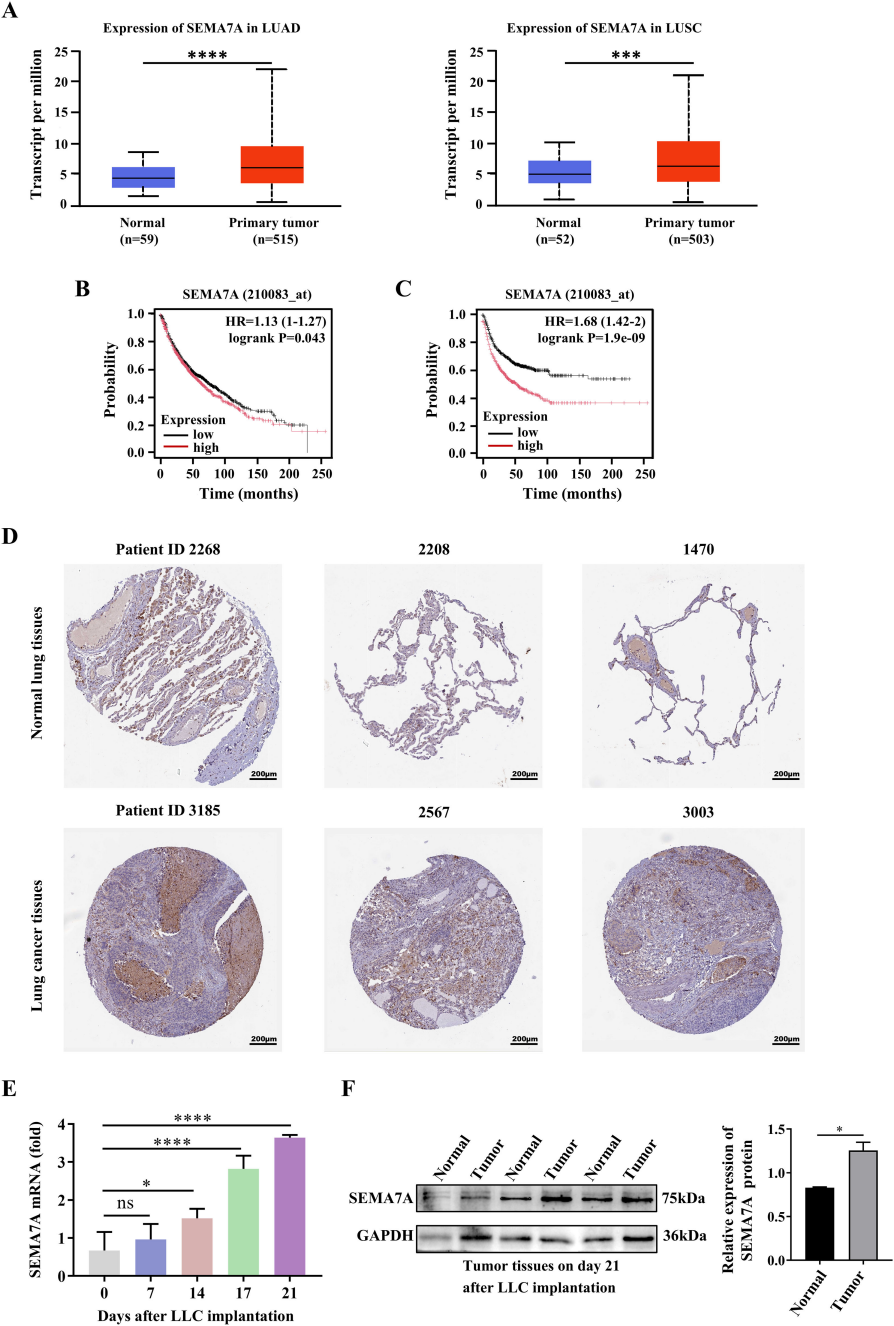
SEMA7A is upregulated in the tumor tissues and is correlated with the tumor poor prognosis. **(A)** The expression levels of SEMA7A in LUAD and LUSC cancer tissues and normal tissues in the TCGA. **(B, C)** Survival analysis with the log-rank test was used to determine the correlation between the expression levels of Sema7A and the OS **(B)** and the PFS **(C)** of the NSCLC patients. **(D)** Representative IHC micrographs of SEMA7A in NSCLC samples and normal samples from the HPA database. Scale bar=200 μm. **(E)** Real-time PCR analysis of Sema7A expression in the tumor tissues of tumor-bearing mice at the indicated time points (0, *n*=3; 7, *n*=3; 14, *n*=3; 17, *n*=3; 21, *n*=3). **(F)** Western blot analysis of SEMA7A protein levels in tumor tissues on day 21 after tumor implantation (Normal, *n*=3; Tumor, *n*=3). Data are presented as mean ± SD. *p < 0.05, *** *p* < 0.001, ****p < 0.0001 by Student’s *t* test (**Figures 1A, F**) or one-way ANOVA test (**Figure 1E**). ns=not statistically significant.

To evaluate SEMA7A expression in the NSCLC, we examined the expression of SEMA7A in human tumor tissues and non-carcinoma adjacent tissues, using the TCGA databases. The results showed that the expression levels of SEMA7A were significantly elevated in LUAD and LUSC tumor tissues compared with normal tissues (**Figure 1A**). Log-rank survival analysis revealed that NSCLC patients with higher SEMA7A expression had poorer overall survival (OS) and progression free survival (PFS) rates than those with lower SEMA7A expression (**Figures 1B, C**). In order to confirm the higher expression of SEMA7A in NSCLC at the tissue protein level compared to normal breast tissue, we used immunohistochemical results from the HPA database. The results showed that the protein expression level in NSCLC tissue was significantly higher than in normal tissue. Typical microphotographs of IHC are shown in **Figure 1D**. In addition, a progressively increased expressions of SEMA7A mRNA were detected in the tumor tissues of Lewis lung carcinoma (LLC) tumor-bearing mice (**Figure 1E**). Meanwhile, an elevated SEMA7A protein levels were detected in the tumor tissues on day 21 after tumor implantation (**Figure 1F**). These results suggest that SEMA7A might be related to the progression and outcomes of NSCLC.

### 3.2 Knockdown of SEMA7A leads to a suppressed progression of NSCLC

**Figure 2:**
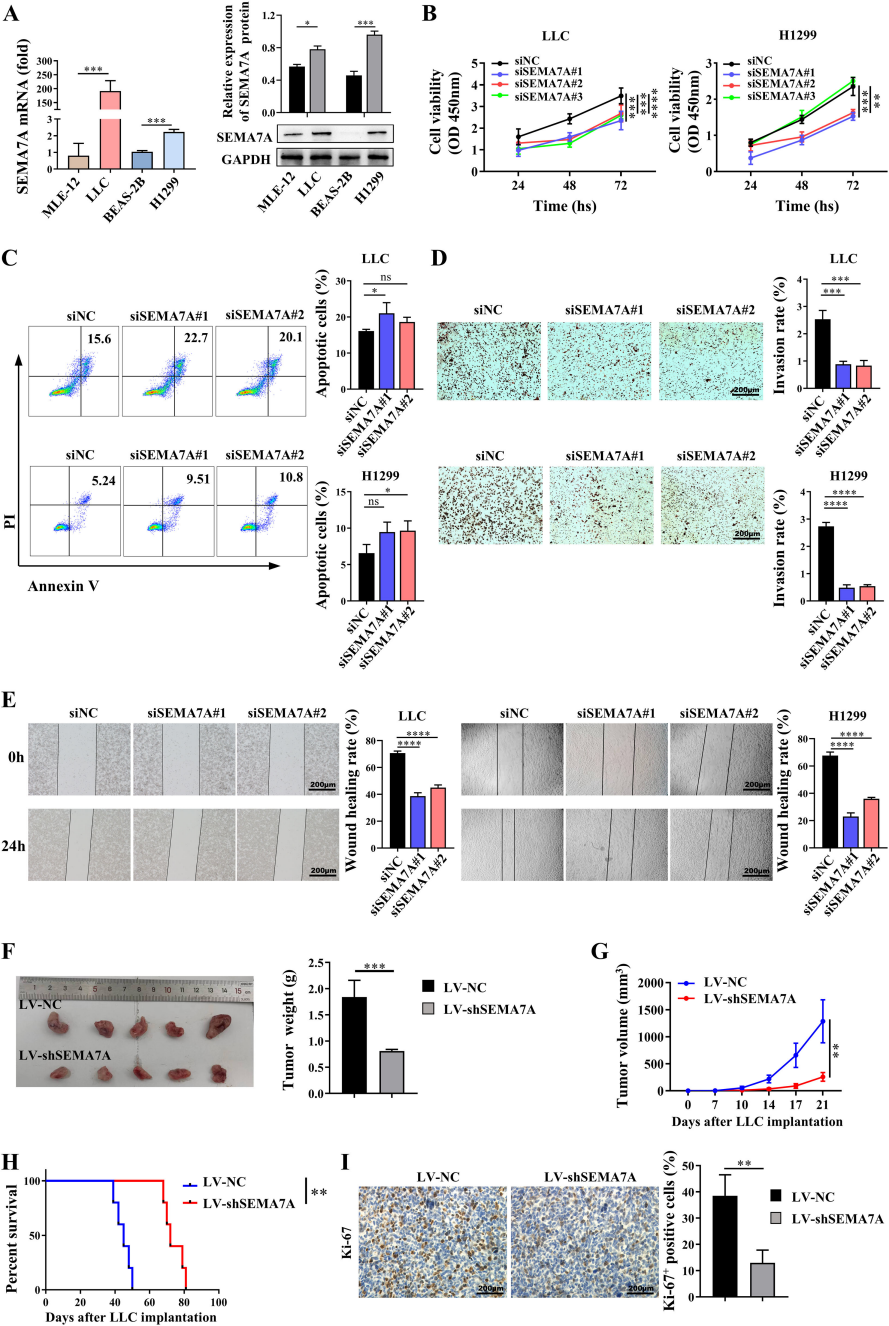
SEMA7A contributes to the progression of NSCLC. **(A)** The mRNA and protein levels of Sema7A in NSCLC cell lines and normal lung epithelial cell lines (MLE-12, *n*=3; LLC, *n*=3; BEAS-2B, *n*=3; H1299, *n*=3). **(B–E)**. LLC and H1299 cells were transfected with SEMA7A siRNA or NC and incubated for intervals. The cell viability at 24, 48 and 72 hours **(B)**, as well as the apoptosis rate **(C)**, the invasion capability **(D)** and the migration capability **(E)** at 24 hours were detected by CCK-8, flow cytometry, Transwell and wound healing assay, respectively (siNC, *n*=3; siSEMA7A#1, *n*=3; siSEMA7A#2, *n*=3; siSEMA7A#3, *n*=3). **(F)** Images of tumor tissues and tumor weight from the mice inoculated with SEMA7A-silenced LLC or control LLC on day 21 after tumor implantation. **(G)** Tumor growth curves at the indicated times after LLC implantation (LV-NC, *n*=5; LV-shSEMA7A, *n*=5). **(H)** Survival analysis of tumor-bearing mice by the log-rank test. **(I)** Immunohistochemical staining images and quantification of Ki-67-positive cells in the tumor tissues on day 21 after SEMA7A-silenced LLC or control LLC implantation. Scale bar=200 μm. Data are presented as mean ± SD. *p < 0.05, **p < 0.01, *** *p* < 0.001, ****p < 0.0001 by Student’s *t* test (**Figures 2A, F–I**), one-way ANOVA test (**Figures 2C–E**) and two-way ANOVA test (**Figure 2B**). ns=not statistically significant.

To confirm whether SEMA7A plays a key role in NSCLC tumor progression, siRNAs were used to silence the SEMA7A gene in the mouse NSCLC cell line LLC and the human NSCLC cell line H1299 (**Supplementary Figures 1A, B**), which have relatively high SEMA7A expression at both the mRNA and protein levels (**Figure 2A**). The results showed that SEMA7A deficiency may attenuate the proliferation but enhance the apoptotic capabilities of LLC and H1299 cells (**Figures 2B, C**). Meanwhile, the invasion and migration activities of these tumor cells were markedly reduced after silencing the SEMA7A gene (**Figures 2D, E**). Moreover, knockdown of SEMA7A in LLC cells resulted in a diminished tumor growth characterized by reduced tumor weight and tumor volume, as well as prolonged survival of the tumor-bearing mice (**Figures 2F–H**). IHC staining also revealed a significant reduction in the proliferative activities of tumor cells in the tumor tissues obtained from the mice implanted with SEMA7A-knockdown-LLC (**Figure 2I**). These experimental results suggest that SEMA7A may contribute to the progression of NSCLC.

### 3.3 Targeting SEMA7A inhibits M2 macrophage polarization

**Figure 3:**
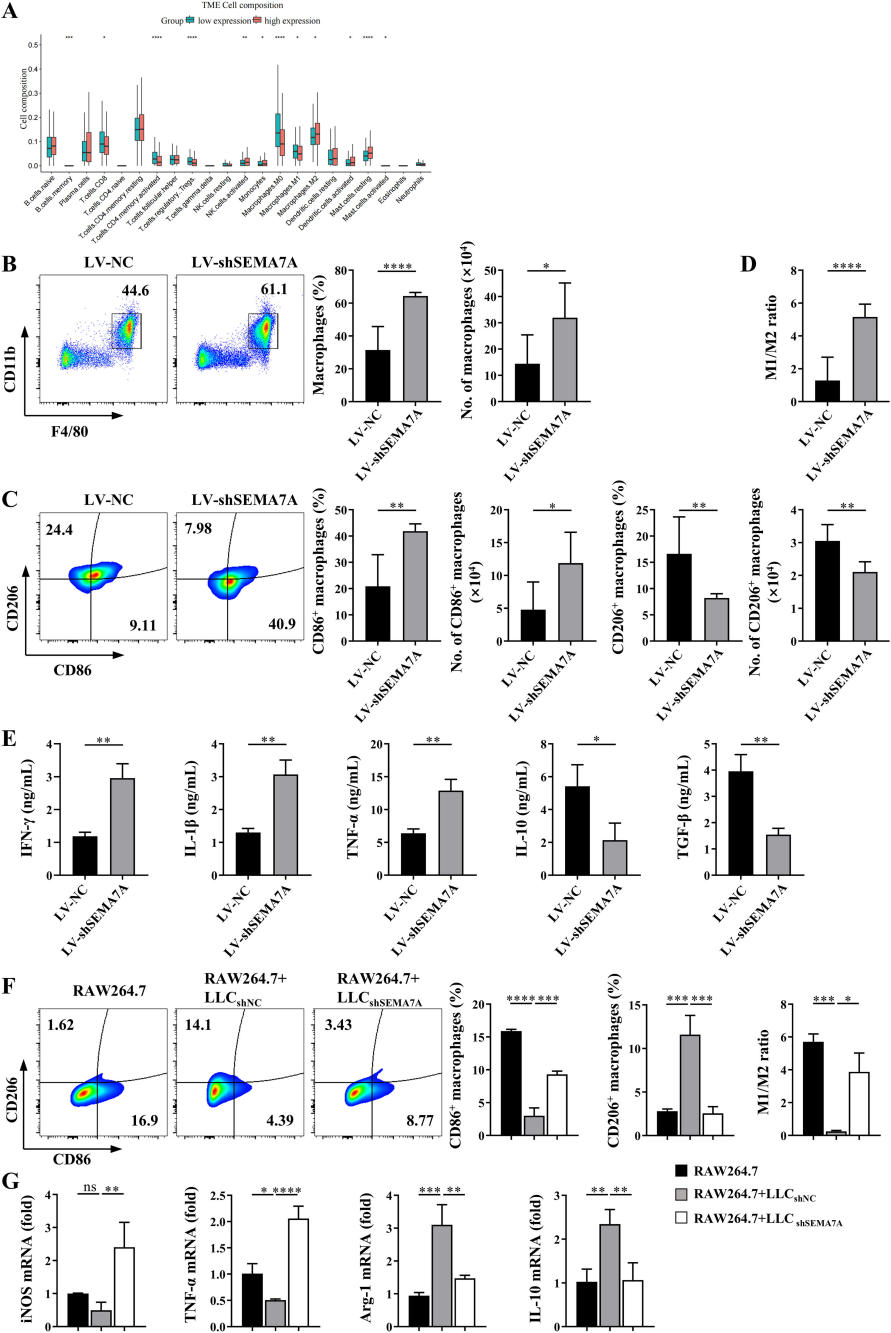
SEMA7A promotes the polarization of TAMs toward M2 phenotype. **(A)** Cell composition in the tumor tissues of NSCLC patients with high (>median level) or low (<median level) SEMA7A expression in the TCGA database. **(B–D)** Tumor tissues were collected from the tumor-bearing mice on day 21 after SEMA7A-silenced LLC or control LLC implantation (LV-NC, *n*=5; LV-shSema7A, *n*=5). The percentages and absolute numbers of total macrophages (CD45^+^CD11b^+^F4/80^+^) **(B)**, M1 (CD45^+^CD11b^+^F4/80^+^/CD86^+^)/M2(CD45^+^CD11b^+^F4/80^+^/CD206^+^) macrophages **(C)** and the M1/M2 ratio **(D)** were detected by flow cytometry. **(E)** The levels of IFN-γ, IL-1β, TNF-α, TGF-β and IL-10 in the tumor tissues of the mice were detected by ELISA. **(F, G)** RAW264.7 cells were cocultured *in vitro* with SEMA7A-silenced LLC or control LLC cells for 24 h (RAW264.7, *n*=3; RAW264.7+LLC_shNC_, *n*=3; RAW264.7+LLC_shSEMA7A_, *n*=3). The percentages of CD86^+^ and CD206^+^ macrophages as well as the M1/M2 ratio were detected by flow cytometry **(F)**. Meanwhile, the expression of the mRNAs for INOS, TNF-α, Arg-1 and IL-10 in the cocultured RAW264.7 cells were measured by real-time PCR **(G)**. Data are presented as mean ± SD. *p < 0.05, **p < 0.01, ***p < 0.001, ****p < 0.0001 by Student’s *t* test (**Figures 3B–E**) or one-way ANOVA test (**Figures 3F, G**). ns=not statistically significant.

By using bioinformatics analysis, we found the levels of SEMA7A might influence the cell composition in the TME of NSCLC (**Figure 3A**). In fact, a significant increase in the percentages and absolute numbers of TAMs, identified as CD11b^+^F4/80^+^ cells, as well as M1-like macrophages, identified as CD11b^+^F4/80^+^CD86^+^ cells, were observed in the TME of tumor-bearing mice inoculated with SEMA7A-knockdown LLC cells (**Figures 3B, C**), but the percentages and absolute numbers of M2-like macrophages, identified as CD11b^+^F4/80^+^CD206^+^ cells were significantly decreased (**Figure 3C**), resulting in an increase in the M1/M2 ratio (**Figure 3D**). In addition, elevated expressions of antitumor factors such as IFN-γ and TNF-a, as well as decreased expression of tumor promoting factors such as IL-10 and TGF-β were detected in the tumor tissues of SEMA7A-knockdown LLC tumor-bearing mice (**Figure 3E**). However, it should be noted that knockdown of SEMA7A did not influence the percentages and absolute numbers of CD4^+^T, CD8^+^T and Treg cells in the TME of tumor-bearing mice (**Supplementary Figures 2A, B**), suggesting that macrophages might be the main target cells of SEMA7A.

To further confirm the effects of SEMA7A on macrophage polarization, coculture of RAW264.7 cells and LLC cells *in vitro* were performed. As shown in **Figure 3F**, the proportion of CD206^+^ macrophages were decreased, whereas the proportion of CD86^+^ macrophages were increased in RAW264.7 cells that were cocultured with SEMA7A-knockdown-LLC cells, compared with that cocultured with the normal LLC cells (**Figure 3F**). Moreover, the mRNA levels of M1 factors, such as iNOS and TNF-α, were remarkably upregulated, whereas the expression levels of M2 factors, such as Arg-1 and IL-10, were downregulated in RAW264.7 cells cocultured with SEMA7A-knockdown-LLC cells (**Figure 3G**). These results suggest that SEMA7A may promote the polarization of TAMs toward M2 phenotype.

### 3.4 SEMA7A affects macrophage polarization in an ITGB1-dependent manner

**Figure 4:**
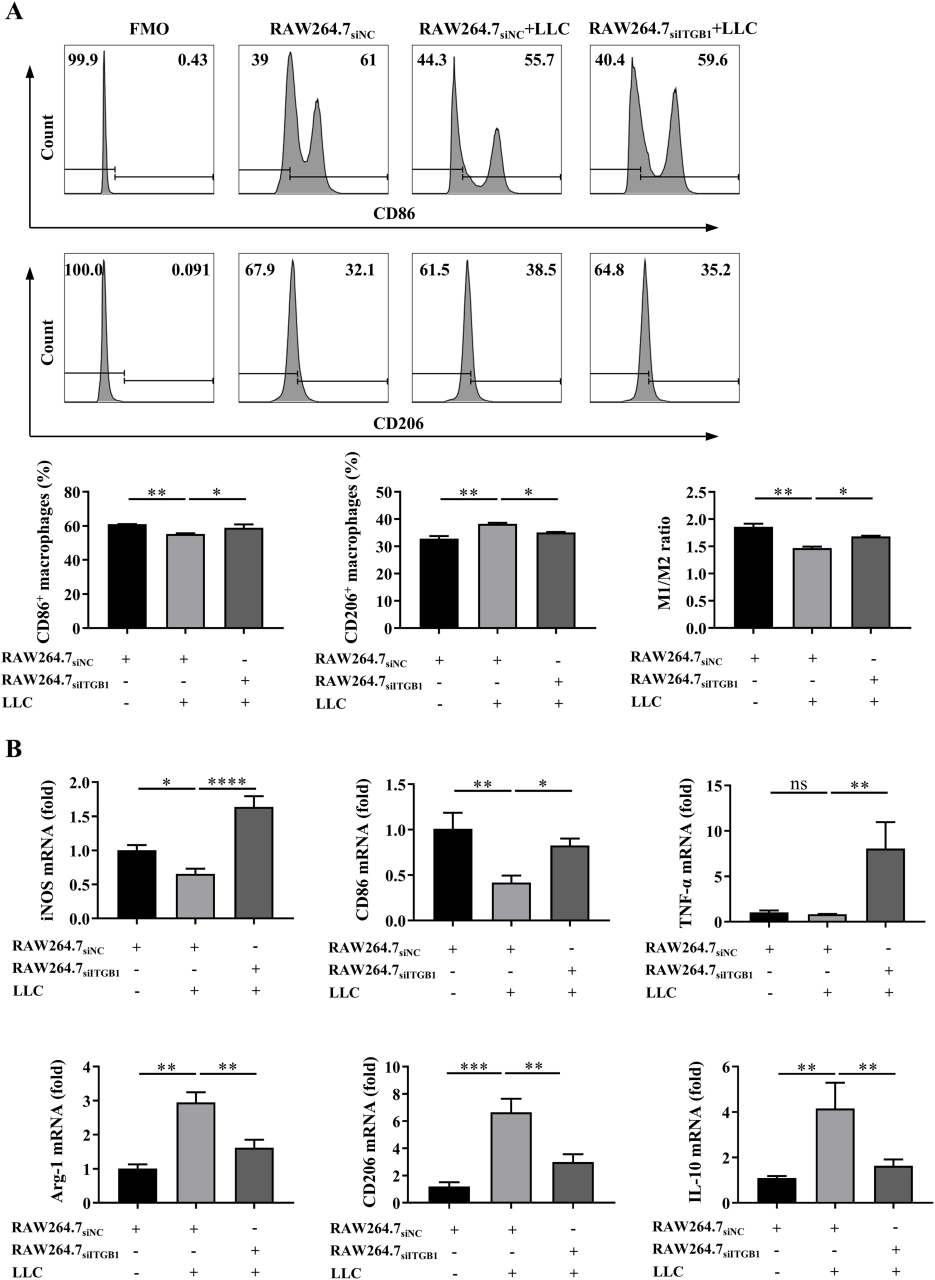
SEMA7A promotes M2 polarization in an ITGB1-dependent manner. **(A, B)** The siITGB1- or NC-transfected RAW264.7 cells were cocultured with LLC cells for 24 h (RAW264.7_siNC_, *n*=3; RAW264.7_siITGB1_, *n*=3; LLC, *n*=3). Then, the percentages of CD86^+^ and CD206^+^ macrophages **(A)**, as well as the expression levels of mRNAs for iNOS, CD206, TNF-α, Arg-1, CD206 and IL-10 in the cocultured RAW264.7 cells **(B)** were detected by flow cytometry or real-time PCR, respectively. Data are presented as mean ± SD. *p < 0.05, **p < 0.01, ***p < 0.001, ****p < 0.0001 by one-way ANOVA test. ns=not statistically significant.

Since SEMA7A exerts its immune function via the receptor integrin β1 (ITGB1) (12) and macrophages express relatively high levels of ITGB1 (26), it is possible that SEMA7A may promotes TAM polarization through the ITGB1 receptor. We found that the coculture of ITGB1-silenced RAW264.7 cells with LLC cells may result in a decrease in the proportion of CD206^+^ macrophages but an increase in the proportion of CD86^+^ macrophages (**Figure 4A**). Furthermore, reduced expressions of mRNAs for CD206, Arg-1 and IL-10 were detected in the ITGB1-silenced RAW264.7 cells (**Figure 4B**), suggesting that SEMA7A may promote M2 polarization in an ITGB1-dependent manner.

### 3.5 Fatty acid oxidation contributes to SEMA7A-promoted M2 polarization in the TME of tumor-bearing mice

**Figure 5:**
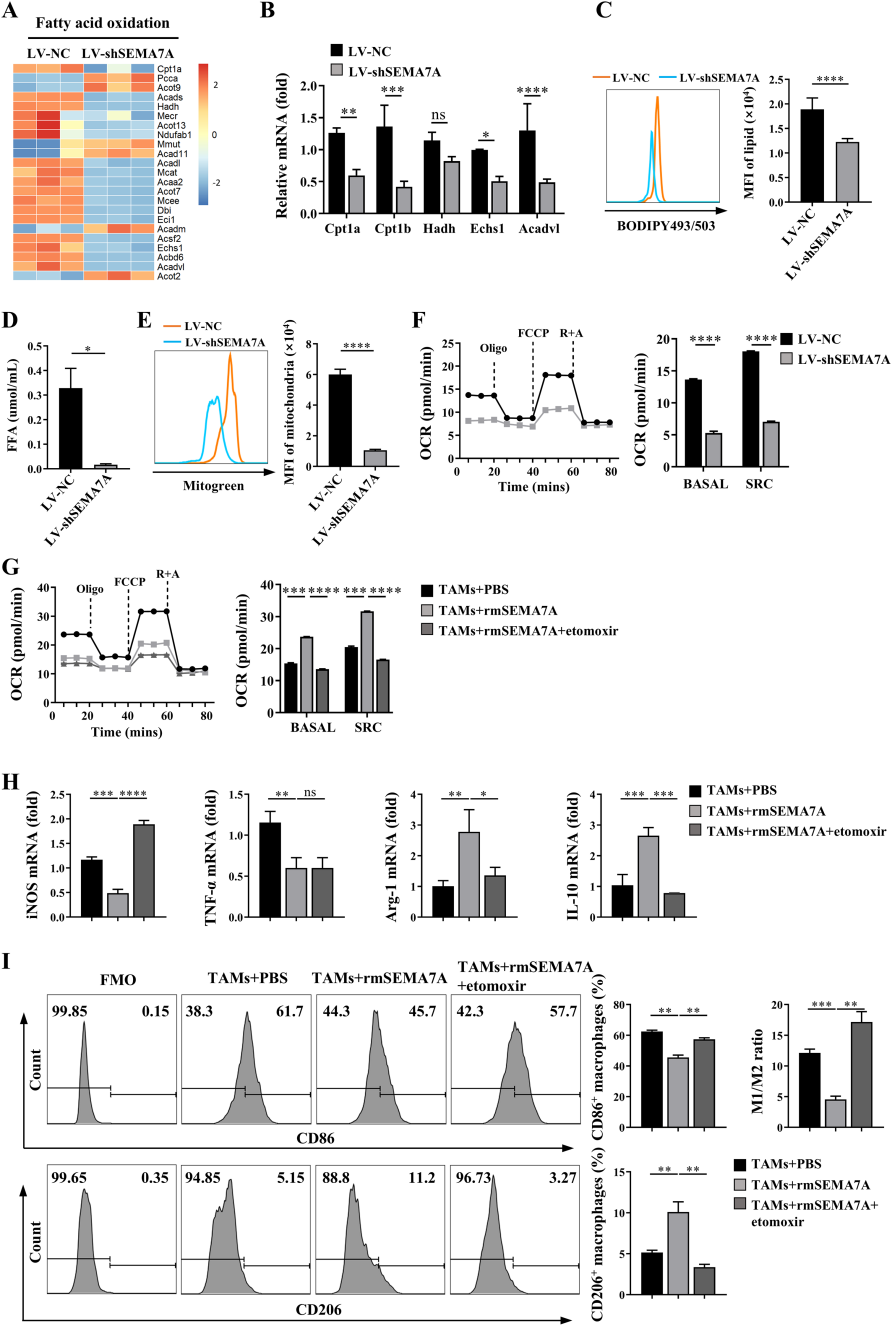
Fatty acid oxidation is essential for SEMA7A-promoted M2 polarization in the TME of tumor-bearing mice. TAMs were sorted from the tumor tissues of the mice on day 21 after SEMA7A-silenced LLC or control LLC implantation (LV-NC, *n*=5; LV-shSEMA7A, *n*=5). **(A)** RNAseq analysis of the expression of FAO-related genes in the sorted TAMs. **(B)** Real-time PCR analysis of the expression levels of mRNAs for FAO-related genes. **(C)** Flow cytometry analysis of the lipid accumulation in the sorted TAMs. **(D)** The concentration of FFAs in the sorted TAMs was measured with a detection kit. **(E)** The quantity of mitochondria in the sorted TAMs was determined by flow cytometry analysis. **(F)** The OCR in the sorted TAMs was detected by using an O2k-FluoRespirometer. **(G–I)** The sorted TAMs were incubated with the recombinant SEMA7A protein in the presence or absence of the FAO inhibitor etomoxir for 24 h (TAMs+PBS, *n*=3; TAMs+rmSEMA7A, *n*=3; TAMs+ rmSEMA7A+etomoxir, *n*=3). Then, the OCR **(G)**, the relative expressions of mRNAs for iNOS, TNF-α, Arg-1 and IL-10 **(H)**, as well as the percentages of CD86+ and CD206+ macrophages **(I)** were detected. Data are presented as mean ± SD. *p < 0.05, **p < 0.01, ***p < 0.001, ****p < 0.0001 by Student’s t test (**Figures 5B–F**), one-way ANOVA test (**Figures 5G–I**). ns=not statistically significant.

Increasing evidence highlights the crucial role of metabolic reprogramming in macrophage activation (29). Therefore, it is necessary to clarify whether SEMA7A-associated M2 polarization may be related to the metabolic reprogramming in the macrophages. Then, we analyzed the expression of genes involved in lipid and glucose metabolism in TAMs sorted from the tumor tissues of the mice on day 21 after SEMA7A-silenced LLC or control LLC implantation. The results showed that most of the genes involved in fatty acid oxidation (FAO) were downregulated in the TAMs sorted from the SEMA7A-silenced LLC-bearing mice compared with those sorted from the control LLC tumor-bearing mice (**Figures 5A, B**). Notably, no significant differences in the genes related to glucose metabolism were detected in these TAMs (**Supplementary Figures 3A–C**), suggesting that fatty acid oxidation (FAO) might be an important pathway for SEMA7A-mediated M2 polarization. In fact, a decreased lipid accumulation (**Figure 5C**) and a lower concentration of free fatty acid (FFA) (**Figure 5D**) were observed in the TAMs isolated from the tumor tissues of SEMA7A-silenced LLC-bearing mice. In addition, the quantity of mitochondria in these TAMs was limited (**Figure 5E**), followed by a decrease in the mitochondrial oxygen consumption rate (OCR) (**Figure 5F**). Blockade of FAO by treatment of the sorted TAMs with etomoxir, an inhibitor of a rate-limiting enzyme of mitochondrial fatty acid oxidation-CPT1A, may restore the reduced OCR caused by knockdown of SEMA7A in the TAMs (**Figure 5G**). Moreover, blocking FAO may significantly decrease the expression levels of mRNAs for M2 factors such as Arg-1 and IL-10 but increase the levels of mRNAs for M1 factor iNOS (**Figure 5H**). In addition, the percentage of M2 macrophages was decreased but the proportion of M1 macrophages was increased, resulting in an elevated M1/M2 ratio after treatment of SEMA7A protein-stimulated TAMs with etomoxir (**Figure 5I**). Together, these results indicate that FAO may be essential for SEMA7A induced M2 polarization in the TME of tumor-bearing mice.

### 3.6 The MAPK/ERK1/2 pathway is involved in SEMA7A-regulated fatty acid oxidation in M2 macrophages

**Figure 6:**
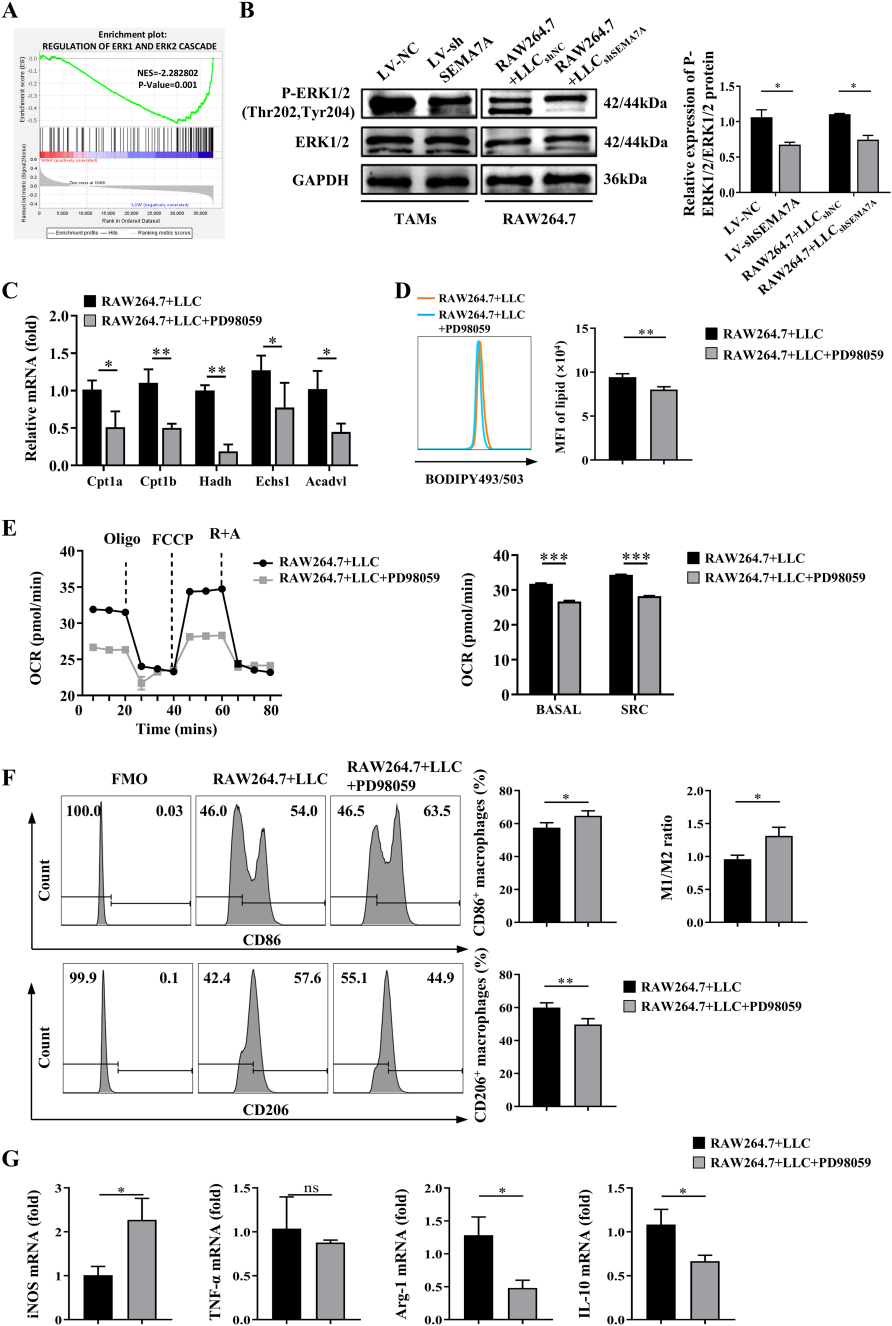
The ERK1/2 signaling pathway contributes to SEMA7A-regulated fatty acid oxidation in macrophages. **(A)** GSEA enrichment analysis of the correlation between SEMA7A and the ERK pathway in the TAMs sorted from the tumor tissues of the SEMA7A-silenced LLC-bearing mice or control LLC-bearing mice on day 21 after tumor implantation. **(B)** Western blot analysis of the protein levels of ERK1/2 in the TAMs, sorted from the SEMA7A-silenced LLC- or control LLC-bearing mice on day 21 after tumor implantation (LV-NC, *n*=5; LV-shSEMA7A, *n*=5), and the RAW264.7 cells cocultured with LLC for 24 h (RAW264.7+LLC_shNC_, *n*=3; RAW264.7+LLC_shSEMA7A_, *n*=3). **(C–G)** The RAW264.7 cells were cocultured with LLC cells for 24 h in the presence or absence of the ERK1/2 inhibitor PD98059 (RAW264.7+LLC, *n*=3; RAW264.7+LLC+PD98059, *n*=3). The mRNA levels of FAO-related genes **(C)**, lipid droplets **(D)**, the mitochondrial OCR **(E)**, and the percentages of CD86^+^ and CD206^+^ macrophages **(F)**, as well as the mRNA levels of iNOS, TNF-α, Arg-1 and IL-10 **(G)** in the cocultured RAW264.7 cells were determined by flow cytometry or real-time PCR. Data are presented as mean ± SD. *p < 0.05.

By performing GSEA, we found that the ERK1 and ERK2 signaling pathway might be related to the expression levels of SEMA7A (**Figure 6A**). In fact, the protein levels of phosphorylated ERK1/2 were significantly lower in the TAMs sorted from SEMA7A-silenced LLC-bearing mice than in those sorted from control LLC-bearing mice (**Figure 6B**). *In vitro* coculture experiments further confirmed that knockdown of SEMA7A may reduce the LLC-induced increase in phosphorylated ERK1/2 proteins in the cocultured RAW264.7 cells (**Figure 6B**). Blockade of the ERK1/2 pathway with the inhibitor PD98059 may decrease the expression levels of mRNAs for FAO-related genes in the RAW264.7 cells cocultured with LLC cells (**Figure 6C**). Besides, the accumulation of lipid droplets and the mitochondrial OCR in the cocultured RAW264.7 cells were also reduced after blocking the ERK1/2 pathway in the cells (**Figures 6D, E**). Notably, treatment of the cocultured RAW264.7 cells with the inhibitor PD98059 may decrease the percentages of CD206^+^ macrophages and the expression levels of mRNAs for M2 markers, such as Arg-1 and IL-10, but increase the percentages of CD86^+^ macrophages and the expression levels of mRNAs for M1 markers, such as INOS, in the cocultured RAW264.7 cells (**Figures 6F, G**). Taken together, these results suggest that SEMA7A may regulate fatty acid oxidation in the macrophages via the ERK1/2 pathway.

## 4 Discussion

SEMA7A, a member of the semaphorin family, is a protein encoded by the SEMA7A gene on chromosome 15 and exists in two forms, either the transmembrane form or soluble form (12). More recently, SEMA7A has been reported to be a protumor factor that promotes the progression of multiple cancers, such as melanoma (17), oral cancer (18), breast cancer (30) and glioma (31) by promoting the proliferation, invasion and migration of tumor cells as well as the epithelial-to-mesenchymal transition. However, to data, little is known about the correlation between SEMA7A and NSCLC. In this study, by using bioinformatics analysis and an experimental murine tumor model, we found that SEMA7A was highly expressed in the tumor tissues of the patients with NSCLC, and the level of SEMA7A was positively correlated with poor survival. Knockdown of SEMA7A can diminish tumor growth, suggesting SEMA7A may be a risk factor for the tumorigenesis and progression of NSCLC. In terms of the underlying mechanisms, the altered polarization status of macrophages in the tumor microenvironment of NSCLC may be responsible for SEMA7A-accelerated tumor progression.

As major components of the tumor microenvironment, tumor-associated macrophages play pivotal roles in tumor growth and metastasis (32, 33). Compared with other types of lung cancer, more TAMs are present in the tumor tissues of NSCLC patients and the polarization status of the TAMs is associated with the relapse-free survival rate and angiogenesis (34). Typically, TAMs can be classified into two subtypes on the basis of their functions: the M1 phenotype has proinflammatory and antitumor capabilities, whereas the M2 phenotype acts as an anti-inflammatory factor and tumor progression promoter (35–37). Although few studies have reported the effects of SEMA7A on the polarization of TAMs, the expression levels of SEMA7A may affect the macrophage polarization induced by Bacteroides fragilis in the gut in the context of type 2 diabetes (38). In addition, SEMA7A can trigger macrophages toward an anti-inflammatory phenotype that inhibits the development of colitis (26). In this study, we found that the knockdown of SEMA7A in the tumor cells resulted in a decrease in the percentage of M2-like macrophages but an increase in the percentage of M1-like macrophages, suggesting that SEMA7A may have regulatory effects on TAM polarization in the TME of lung tumor-bearing mice.

At present, SEMA7A is believed to exert its effects by binding to the functional receptor plexin C1 or integrin receptors containing the β1 subunit (β1 integrin). Under our experimental conditions, SEMA7A seems to regulate TAM polarization via the receptor β1 integrin, since silencing the expression of β1 integrin in macrophages may diminish the regulatory effects of SEMA7A on macrophage polarization. In fact, unlike other members of the SEMA family, SEMA7A uses mainly β1 integrins as receptors in both the nervous and immune systems (10, 39). For example, recombinant SEMA7A protein can stimulate monocytes/macrophages through β1 integrin to induce proinflammatory cytokine production by these cells (40). Consistent with the literature reports, our experimental results suggest that tumor-derived SEMA7A may promote M2 polarization in an β1 integrin-dependent manner.

With respect to the mechanisms by which SEMA7Aalters macrophage polarization, the reprogramming of intracellular metabolism has been a focus (41, 42). It has been reported that lipopolysaccharide (LPS)/IFN-γ-activated M1 macrophages exhibit elevated glycolysis and fatty acid synthesis. In contrast, IL-4/IL-10-activated M2 macrophages display increased fatty acid oxidation (43, 44). In the present study, decreased fatty acid oxidation, but not glucose metabolism, was observed in the TAMs sorted from the tumor tissues of SEMA7A-silenced LLC-bearing mice, suggesting fatty acid oxidation may be required for SEMA7A-mediated M2 polarization. In fact, the inhibition of FAO or lysosomal lipolysis impairs IL-4-induced M2 activation (43). Besides, fatty acid metabolism is essential for receptor-interacting protein kinase 3 deficiency-induced M2 activation (45). Recently, a study revealed that deletion of SEMA7A may reduce oxidative phosphorylation in mouse peritoneal macrophages (28), suggesting that SEMA7A may regulate macrophage polarization by affecting lipid metabolism in the cells. Notably, the ERK1/2 signaling pathway seems to be essential for SEMA7A-regulated fatty acid oxidation, since a decrease in the SEMA7A-mediated lipid metabolism following an enhanced M1 polarization were observed in the TAMs treated with an ERK inhibitor. Really, the MAPK/ERK signaling pathways are key downstream effectors of integrin receptors in multiple lung cancers (46). Moreover, the MAPK signaling pathway may contribute to fatty acid oxidation via the upregulation of carnitine palmitoyl transferase 1A (CPT1A), a key rate-limiting enzyme of fatty acid oxidation (47) which was significantly decreased in the cocultured macrophages treated with an ERK inhibitor (**Figure 6**).

In summary, this study revealed a correlation between the levels of SEMA7A and the outcomes of NSCLC. SEMA7A may promote M2 polarization by affecting fatty acid oxidation in cells in an ITGB1-dependent manner to promote NSCLC progression. SEMA7A/ITGB1-triggered ERK1/2 activation seems to be necessary for SEMA7A-regulated fatty acid oxidation during the M2 polarization.

## Data Availability

The original contributions presented in the study are included in the article/**Supplementary Material**. Further inquiries can be directed to the corresponding authors.
