# Supplementary material for: Tumor-derived SEMA7A regulates fatty acid oxidation in the tumor-associated macrophages to promote the progression of non-small cell lung cancer
Source: Front Immunol. 2025 Sep 11;16:1625208. doi: 10.3389/fimmu.2025.1625208 (PMC12460325; doi:10.3389/fimmu.2025.1625208)
Supplement: Supplementary file 1 [file DataSheet1.docx]

Supplementary Material

# Supplementary Figures and Tables

For more information on Supplementary Material and for details on the different file types accepted, please see [here](https://www.frontiersin.org/guidelines/author-guidelines#supplementary-material).

## Supplementary Figures


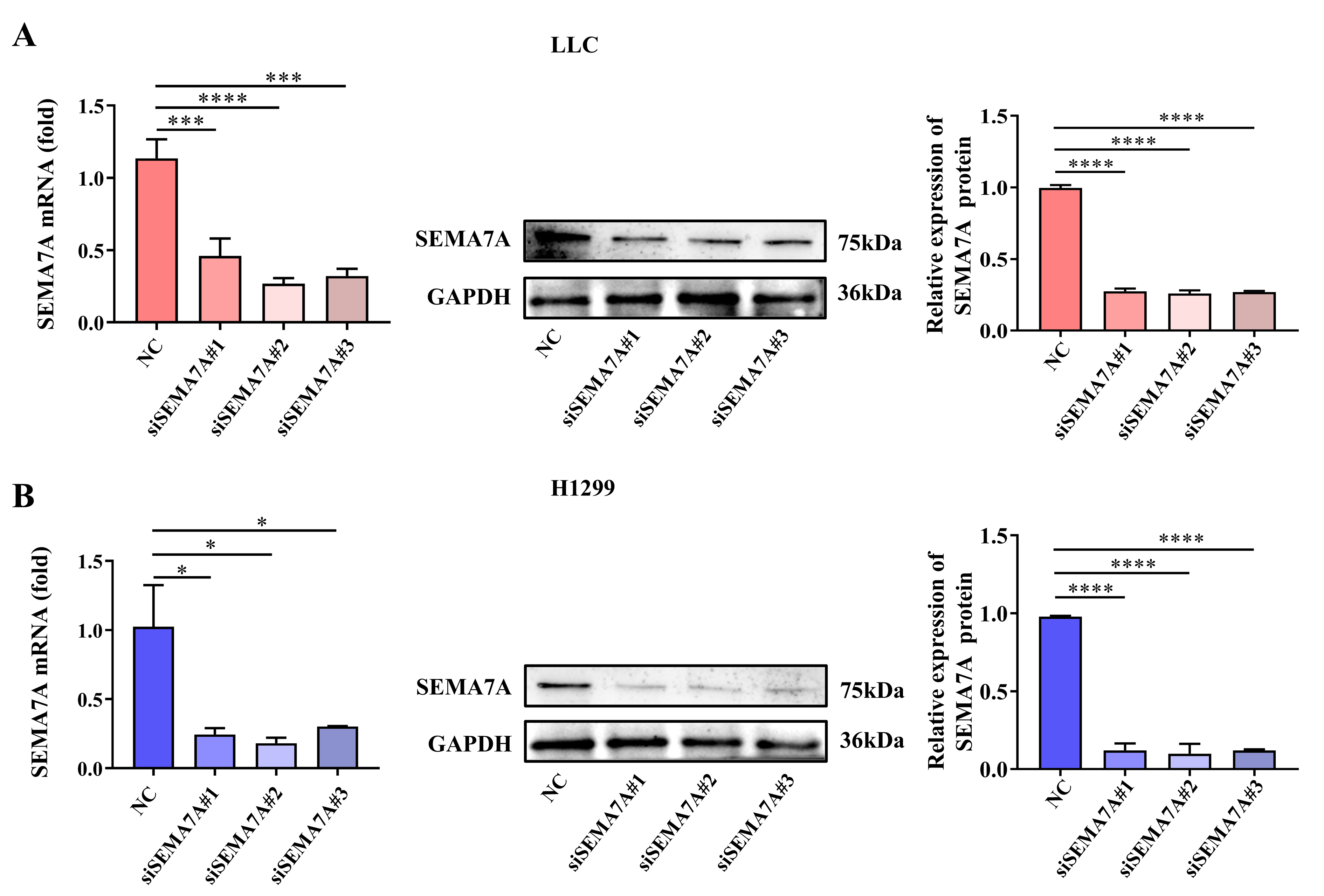


**Supplementary Figure 1.** **LLC and H1299 cells were transfected with SEMA7A siRNA or NC and incubated for 48 hours.**

The mRNA and protein levels of SEMA7A were measured by real-time PCR and western blot in LLC (A) and H1299 (B) cells to verify the knockdown efficiency. Data are presented as mean ± SD. *p < 0.05, *** p < 0.001, ****p < 0.0001 by one-way ANOVA test (Supplementary Fig. 1A, B).


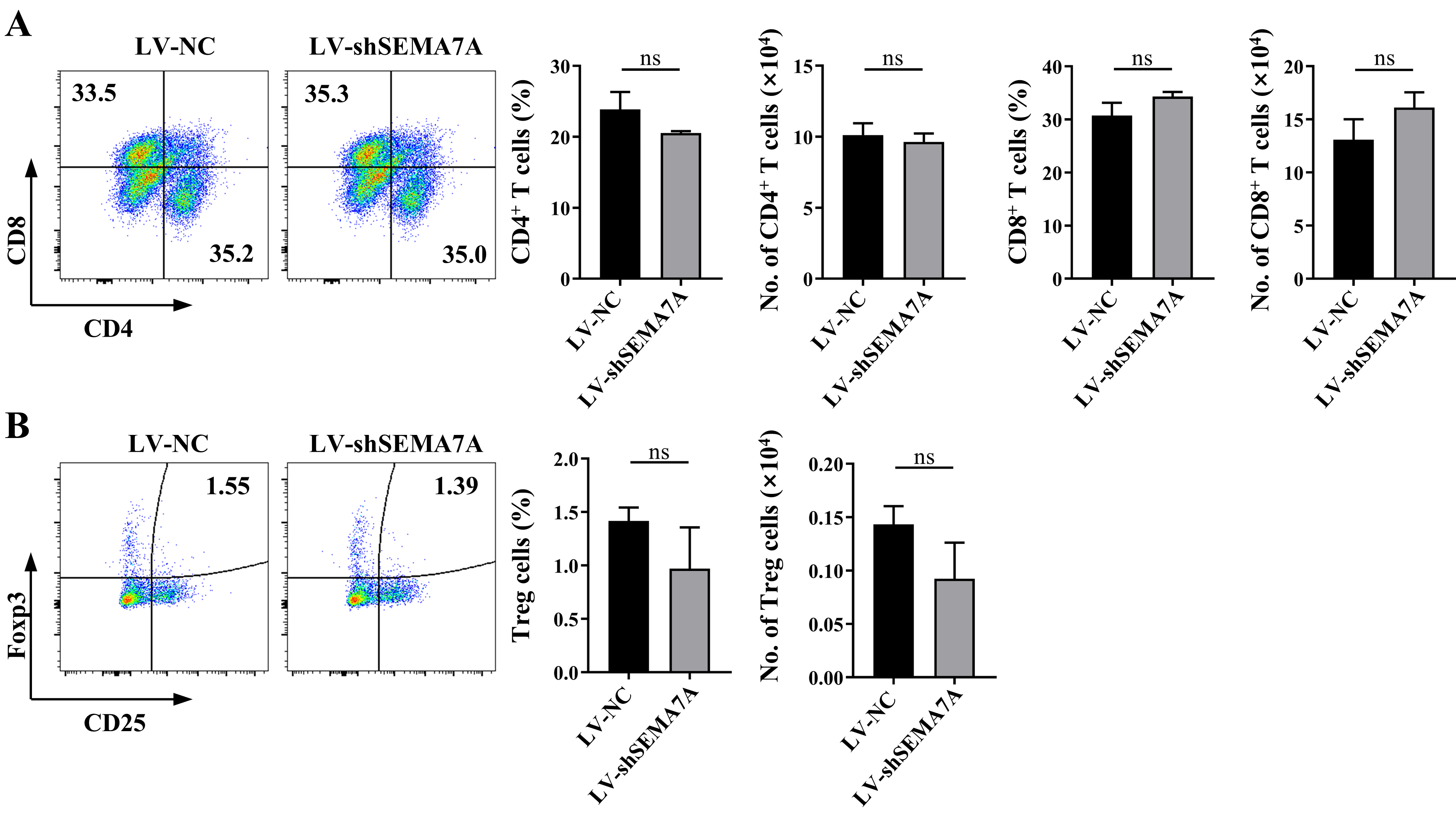


**Supplementary Figure 2.** **Targeting SEMA7A does not affects the percentages and absolute numbers of T cell subtypes in the TME of tumor-bearing mice.**

Tumor tissues were collected from SEMA7A-silenced LLC and control LLC-bearing mice on day 21 after tumor implantation. (A, B) The percentages and absolute numbers of CD4+/CD8+ T cells (A) and Treg cells (B) in the TME were detected by flow cytometry. Data are presented as mean ± SD. Student’s t test was used for analysis of the difference between the two groups. ns=not statistically significant.


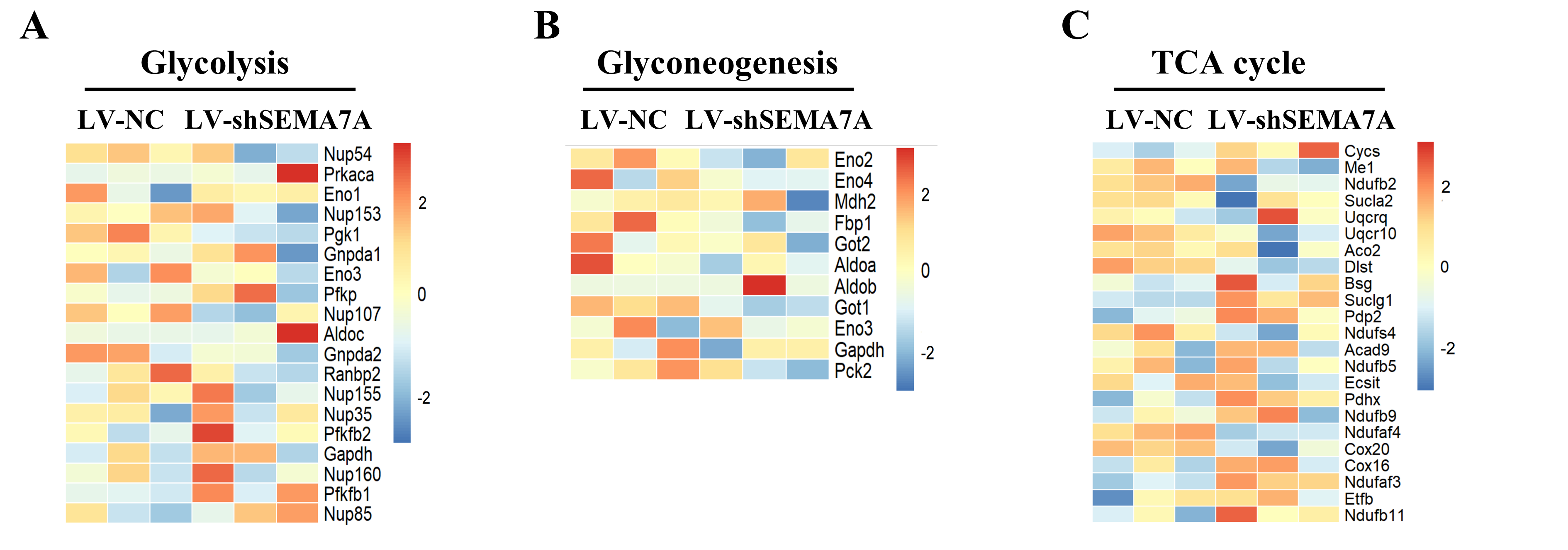


**Supplementary Figure 3. Silencing SEMA7A does not alter the glycolytic metabolism in the macrophages.**

Macrophages were sorted from the tumor tissues of the mice on day 21 after SEMA7A-silenced LLC or control LLC implantation. (A-C) The relative expression levels of genes involved in the process of glycolysis (A), gluconeogenesis (B) and TCA cycle (C) in the sorted TAMs were determined using transcriptome sequencing method.

## Supplementary Tables

**Supplementary Table 1.** The sequences of the designed oligonucleotides against SEMA7A and NC

| **siRNA** | **Sense（5'-3'）** | **Antisense（5'-3'）** |
| --- | --- | --- |
| Scramble(house  mouse) | UUCUCCGAACGUGUC ACG UTT | ACGUGACACGUUCGG AGAATT |
| siSEMA7A(house mouse)-1970 | CCUAGCUGCAUCCUGUUCAUUTT | AAUGAACAGGAUGCAGCUAGGTT |
| siSEMA7A(house mouse)-1348 | CCAUAGCUUUGUCUUCAAUAUTT | AUAUUGAAGACAAAGCUAUGGTT |
| siSEMA7A(house mouse)-709 | CCAAGCCUAUGAUGAUAAGAUTT | AUCUUAUCAUCAUAGGCUUGGTT |
| Scramble(human) | CCUUCGGAACGUGUC ACG UCC | GGCAGACACGUUCGG AGAACC |
| siSEMA7A(human)-1658 | ATGAGAGGCUAGCCC  UTCAGTCCC | CCUAGCUGCAUCCUGUUCAUUTT |
| siSEMA7A(human)-1020 | ATGCCUGGTUCTCAGGAGUCATCCC | CCAUUGAAGACAAAGCUAUGGTT |
| siSEMA7A(human)-870 | ATUCCTGAAGGACUAGUGACATCCC | AUCUUAUCAUCAUAGGCUUGGTT |

**Supplementary Table 2.** Primers used for real-time PCR

| **Gene** | **Forward (5’-3’)** | **Reverse (5’-3’)** |
| --- | --- | --- |
| SEMA7A | GCCACCAACAGGAACTTCAATCGG | TTGGAGAAAACGCCATAGACCCT |
| TNF-α | CGGTGGTGGGACTCGTATG | CTGGTTGTCTTCCAGCTTCACA |
| IL-10 | GCTCTTGCACTACCAAAGCC | CTGCTGATCCTCATGCCAGT |
| iNOS | CAAGCTGAACTTGAGCGAGGA | CAACGAGCGGTTCCGATG |
| Arg-1 | AGCTCTGGGAATCTGCATGG | ATGTACACGATGTCTTTGGCAGATA |
| CD86 | CTTACGGAAGCACCCACGAT | TGTAAATGGGCACGGCAGAT |
| CD206 | GTCAGAACAGACTGCGTGGA | AGGGATCGCCTGTTTTCCAG |
| Cpt1a | TGGCATCATCACTGGTGTGTT | GTCTAGGGTCCGATTGATCTTTG |
| Cpt1b | GCACACCAGGCAGTAGCTTT | CAGGAGTTGATTCCAGACAGGTA |
| Hadh | TCAAGCATGTGACCGTCATCG | TGGATTTTGCCAGGATGTCTTC |
| Echs1 | AGCTATACCGTTTGCCACCC | GCACCTGCCCTTACAGTGAA |
| Acadvl | CTACTGTGCTTCAGGGACAAC | CAAAGGACTTCGATTCTGCCC |
| β-actin | TCCATCATGAAGTGTGACGT | TACTCCTGCTTGCTGATCCAC |
| hSEMA7A | CGCCGGCTCGGTTGG | TGTAGTTCTCGCAGTCCGTG |

**Supplementary Table 3.** Antibodies used in the flow cytometry staining

| **Antibody** | **Catalog number** | **Manufacturer** |
| --- | --- | --- |
| FITC anti-CD45 | 103107 | BioLegend |
| BV421 anti-CD11b | 101251 | BioLegend |
| APC anti-F4/80 | 123116 | BioLegend |
| PE-CY7 anti-CD86 | 105013 | BioLegend |
| PE anti-CD206 | 141705 | BioLegend |
| FITC anti-CD3 | 100204 | BioLegend |
| APC-CY7 anti-CD4 | 100413 | BioLegend |
| BV510 anti-CD8 | 563068 | BioLegend |
| PE anti-Foxp3 | 320007 | BioLegend |
| APC anti-CD25 | 102011 | BioLegend |
| BODIPY493/503 | GC42959 | Glpbio |
| Mito-Tracker Green | C1048 | Beyotime |
